# Sustainable road network design considering hydrogen fuel cell vehicles

**DOI:** 10.1038/s41598-023-49264-1

**Published:** 2023-12-11

**Authors:** Hongxi Liu

**Affiliations:** Section of Foreign Languages, Department of Basic Studies, Criminal Investigation Police University of China, Shenyang, 110854 China

**Keywords:** Civil engineering, Environmental impact

## Abstract

Environmental pollution and energy shortages have brought about an increased focus on new energy vehicles. Hydrogen fuel cell vehicles (HFCVs) have experienced rapid development due to the potential to alleviate energy pressures and reduce pollution emissions. Near zero-carbon emissions offer a promising avenue for promoting sustainable transportation development. To evaluate the impact of HFCVs on the transportation environment, this paper investigates the problem of sustainable transportation network design including HFCVs. Specifically, the problem is formulated as a bi-level multi-objective programming problem, with the upper level aimed at determining the optimal network design scheme considering multiple objectives, while the lower level addresses the mixed traffic flow that comprises both HFCVs and fuel vehicles. To solve the multi-objective sustainable network design problem, an integrated solution framework that combines the particle swarm optimization (PSO) algorithm with the Frank-Wolfe algorithm (FW) is developed. Specifically, the PSO algorithm is utilized to solve the upper-level model and identify the optimal network design schemes, while FW algorithm is adopted to handle mixed traffic flow assignments. Finally, the proposed model and algorithm are implemented in two numerical experiment to demonstrate their effectiveness and efficiency.

## Introduction

Hydrogen Fuel Cell Vehicles (HFCVs) are rapidly gaining attention because of the ability to mitigate the energy crisis and promote the sustainable transportation development^1–3^. HFCVs are considered an eco-friendly transportation travel modal that can contribute to achieving sustainable transportation goals by reducing CO_2_ emissions. As a result, HFCVs have garnered significant attention from the government, industry, and academia^4,5^. Many automobile manufacturers are investing considerable resources in HFCV research and development^6,7^. Actually, HFCVs have been applied in certain specific events, such as during the 2022 Beijing Winter Olympic Games, in which were used to transport athletes and participants^8^. In the future, with advances in HFCV technology, there will be two types of traffic flow on urban roads: fuel vehicles (FVs) and HFCVs^9^. In this context, it will be important for traffic planners to analyze the transportation environmental impacts when performing a transportation network design.

Transportation network design problems that consider environmental impacts can be categorized as a sustainable network design problem (SNDP)^10,11^. By seeking the optimal road network design scheme based on traffic assignment to achieve the simultaneous optimization of traffic efficiency and traffic environment. However, the perception travel costs of HFCV users differs from FV users due to higher environmental awareness. The heterogeneity of HFCVs and FVs leads a new type mixed traffic flow. As a result, transportation planners must consider a mixed traffic flow consisting of HFCVs and FVs, called as SNDP with HFCVs^12^.

This study proposes a multi-objective bi-level programming model to address the SNDP considering HFCVs. The upper model aims to obtain the optimal network design scheme by considering travel costs, CO_2_ emissions, and road construction cost. The lower model is expressed as a multi-class user equilibrium. A comprehensive solution algorithm that combines a meta-heuristic algorithm and Frank-Wolfe is designed to solve the model. Specifically, a multi-objective meta-heuristic algorithm, multi-objective PSO (MPSO), is developed to gain the Pareto frontiers^13^. Meanwhile, a diagonalized Frank-Wolfe algorithm is adopted to handle multi-class user equilibrium^14^. In summary, the main contributions are as follows:

1. We present a Sustainable Network Design Problem (SNDP) considering mixed traffic flow consisting of HFCVs and FVs. SNDP focuses on the impact of HFCV emergence and heterogeneous traffic flow on road network design.
2. A multi-objective bi-level model with multi-class user equilibrium is proposed. The model considers system travel time, CO_2_ emission, and lanes construction cost as the optimal objectives, and the multiclass user equilibrium depicts the travel choice behavior of FVs and HFCVs.
3. A comprehensive solution algorithm is developed to handle the multi-objective bi-level model. The algorithm combines MPSO and the diagonalized Frank-Wolfe, where MPSO is used to address the multi-objective problem, and the diagonalized Frank-Wolfe algorithm is employed to solve the multiclass user equilibrium.

Furthermore, the developed model and algorithm are implemented into two numerical experiments to demonstrate effectiveness and efficiency. The results indicate that total CO_2_ emission costs and total travel costs will decreased based on network design and the emergency of HFCV. The sensitivity analysis shows that as the HFCV penetration rate increases, total travel costs and CO_2_ emissions decrease. Additionally, higher vehicle speed and travel demand also decrease emissions and total travel costs. In conclusion, the development of HFCVs and reasonable road network design can contribute to sustainable transportation development.

The remainder of this paper is structured as follows: section “Literature review” provides a review of the relevant literature from two aspects. Sections “Problem formulation” and “Solution algorithm” describe the research problem and mathematical programming model in detail, respectively. Section “Solution algorithm” elaborates on the proposed solution algorithm. Section “Numerical experiments” presents the numerical experiments, and section “Conclusions” concludes the paper and suggests future research directions.

## Literature review

### Studies on HFCV

Numerous studies demonstrate that hydrogen fuel cell vehicles (HFCVs) are poised to effectively address fuel energy scarcity and diminish CO_2_ emissions^15^. Some scholars have analyzed the development trend of hydrogen fuel cell vehicles from a macro perspective. For example, Bethoux elaborated the composition of HFCV driving system and the advantages of HFCV vehicles, and listed the current research status of HFCV^16^. Offer et al.^17^ conducted a comparative analysis of the advantages, disadvantages, and future development trends of hydrogen fuel cell vehicles, electric vehicles, and hybrid vehicles combining electric and hydrogen fuel, and pointed out that in the future transportation system, hydrogen fuel cell vehicles have more advantages. Zhao et al., based on the supply chain perspective, analyzed the development of hydrogen fuel cells in China from three aspects: policy support, market application, and technological development. The result is that the development of hydrogen production technology and fuel cell technology is crucial for the development of hydrogen fuel cell vehicles^18^. Tao et al. investigated the issue of integrated planning between a hydrogen fuel cell vehicle transportation system and an electric power system that includes electric vehicles. And an optimization model also is proposed for this problem, which considers factors such as the number of hydrogens refueling stations and the penetration rate of hydrogen fuel cells and electric vehicles^19^.

Another body of relevant studies is about the collaborative planning of HFCV and transportation system, Sara Evangelisti et al. propose an all-encompassing evaluation method for fuel cell vehicles that emphasizes the production process and compares it with the production, utilization, and end-of-life processes of both pure electric vehicles and conventional fuel vehicles. This analysis indicates that the reduction of the environmental impact of the manufacturing phase of HFCVs remains a challenge^15^. Pouria Ahmadi et al. examine the environmental impacts and economic costs of switching from conventional gasoline vehicles to HFCVs in four Canadian provinces and demonstrate that HFCVs have reduced life-cycle GHG emissions and lower fuel costs when compared to conventional fuel vehicles^20^. Cai et al. examine the whole-life costs of battery electric vehicles (BEVs), plug-in hybrid electric vehicles (PHEVs), and fuel cell electric vehicles (FCEVs) in China. When compared to conventional gasoline vehicles, the whole-life costs of BEVs, PHEVs, and FCEVs are roughly 1.5 times, 0.5 times, and 2.3 times higher than those of conventional vehicles in the short term, respectively^21^. Li et al. conduct a life cycle cost (LCC) analysis of hydrogen systems utilizing low-cost electricity, in addition to a sensitivity analysis of renewable energy and VE hydrogen production systems in China. The study results indicate that reducing equipment costs, enhancing hydrogen purity to increase retail prices, and improving electrolysis efficiency is vital^22^. Sun et al. develop an HSS (Hydrogen station siting) location optimization model considering hydrogen source planning and hydrogen station planning. The model aims to optimize the number and location of stations simultaneously^23^. Ogden et al. investigate the "social life-cycle cost" of automobiles and conclude that hydrogen-fueled vehicles have the lowest cost and are the future of the automotive industry^24^. However, current studies neglect the impact of HFCVs on emissions concerning transportation network design. In other words, there few studies have looked at transport networks in tandem with hydrogen fuel cell vehicles.

### Sustainable road network design

Sustainable road network design is another area of focus in transportation research, with many studies aimed at improving sustainable transportation through the use of sustainable network design^25^. For example, Lin et al.^6^ propose a sustainable network design problem for connected and autonomous vehicle lanes, using a multi-objective bi-level mathematical model. Wang et al.^26^ investigate the problem of excessive noise in sustainable network design, and propose a paradoxes problem for network design. Sharma and Mathew^27^ develop a bi-level programming model that evaluates the trade-off between system travel time and emissions, with the aim of minimizing both. Ma et al. develop a dynamic traffic assignment model to explore the problem of system efficiency and emission pricing. Amirgholy et al.^28^ also establish a bi-level model that captures the trade-off between total travel time and CO_2_ emissions, using a multi-attribute decision method and diagonalization algorithm. Wang et al.^29^ investigate the bi-modal sustainable network design problem, which considers equity, economy, and environmental factors and use the PSO algorithm to solve the model. For more discussion on sustainable transportation, please refer to^30,31^.

There is also a lot of discussion about hydrogen fuel cell vehicles and sustainable transportation. For example, according to the analysis and comparison of the CO_2_, SO_2_, and other pollutants emitted by traditional fuel vehicles, electric vehicles, and hydrogen fuel cell vehicles, Acar and Dincer^32^ concludes that hydrogen fuel cell vehicles are the best choice for achieving sustainable transportation development. Mierlo et al. has conducted research on the contributions of electric vehicles, hybrid vehicles, and hydrogen fuel cell vehicles to sustainable development in the contexts of passenger cars and trucks. The research findings suggest that hydrogen fuel cell vehicles are a viable development vision in the face of future energy shortages^33^. Ajanovic et al. studied the economic issues related to the development of hydrogen fuel cell vehicles, specifically how to address the problem of high costs through the implementation of reasonable policies. The research also highlights the promising prospects of using hydrogen fuel cell vehicles in public buses^9^. Halder et al. conducted a detailed analysis of the challenges faced in the development of hydrogen fuel vehicles in the context of sustainable transportation. The study pointed out the issues and technological bottlenecks associated with the entire process of hydrogen fuel production and application^34^. However, none of these existing studies have addressed the impact of HFCVs on traffic flow. Therefore, it is necessary to analyze the new traffic situation from a network perspective.

## Problem formulation

Sustainable Road Network Design considering hydrogen fuel cell vehicles is a classical discrete sustainable network design problem (SNDP). SNDP is usually formulated as a bi-level programming model, which the upper is to find the optimal network design scheme and the lower is the multiclass users equilibrium problem. Due to the asymmetric effects of mixed traffic flow, there is no equivalent mathematical programming model for multi-user equilibrium allocation. Hence, we character the multi-user equilibrium problem as nonlinear complementary constraints that equal to the lower level problem. In this section, we first introduce the transportation network notations and basic link travel time function (section “Network representation”). Secondly, we develop the optimal objectives equations (the upper level problem) and the nonlinear complementary constraints (the lower level problem). Finally, the bi-level programming model is expressed as a mixed-integer linear program model (section “Optimization model”).

### Network representation

**Figure 1:**
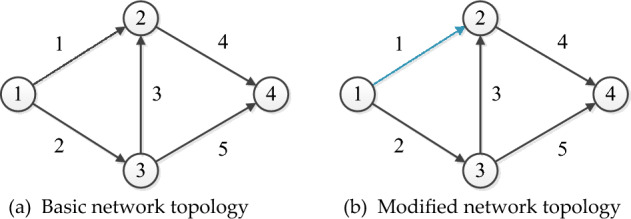
A simple network example.

The design of road networks aims to determine the optimal scheme for road deployment, thereby maximizing the benefits of the network. In this paper, we aim to investigate SNDP with HFCVs to find the optimal road network design scheme. In general, a road network can be represented as a topology comprising nodes and links, as illustrated in Fig. 1a. Let $G(N, A)$ denote the road network, in which $N$ and $A$ denote the set of the nodes and links. $W$ denotes the set of origin–destination pairs, $w\in W$. $R$ denotes the set of travel routes, $r\in R$. Conducting road network design modifies the topology network, which consequently affects travel route selection. For instance, the basic network is presented in Fig. 1a, while the revised network topology is depicted in Fig. 1b, where the blue link 1 represents the realigned road link. Consequently, the capacity of the link changes, and the link travel time differs from that of the basic network. Ultimately, the road network design affects travel route selection.

Without loss of generality, the BRP (Bureau of Public Roads) function is adopted as the link travel time function to character the travel time of link^35^. That is,

1 $$ t_{a} \left( {v_{a} } \right) = t_{0} \left[ {1 + \alpha \left( {\frac{{v_{a} }}{{C_{a} }}} \right)^{\beta } } \right]\quad \forall a \in A $$

where ${t}_{0}$ denotes the free travel time of link $a$; ${v}_{a}$ denotes the traffic volume of link $a$; $\alpha$ and $\beta$ represent the coefficients $\alpha =0.15$, $\beta =4$; ${C}_{a}$ denotes the link capacity, which will change based on the different road network schemes. In detail:

2 $$ C_{a} = \left\{ {\begin{array}{*{20}l} {C_{a} + y_{a} {\mathbb{C}}_{a} } \hfill & {\forall a \in \hat{A}} \hfill \\ {C_{a} } \hfill & {\forall a \in A\backslash \hat{A}} \hfill \\ \end{array} } \right.$$

where ${\mathbb{C}}_{a}$ denotes the increased capacity after link was redesigned; ${y}_{a}$ is the binary variable, if ${y}_{a}$ = 1, the link is selected to be redesigned, otherwise ${y}_{i}$ = 0; $\widehat{A}$ denotes the set of candidate lanes of link $a$. In addition, the route travel time is formulated as follows:

3 $$  t_{{r,w}}^{m}  = \sum\nolimits_{{a \in A}} {\delta _{a}^{{r,w}} t_{a}^{m} } \quad \forall a \in A,m \in M  $$

where ${\delta }_{a}^{r,w}$ is the 0–1 link-path incidence indicator; $m$ denotes the set of travel modes, $m\in M$ = {HFCV, FV}. The cumulative traffic flow of link $a$ is formulated as:

4 $$ \begin{array}{*{20}c} {v_{a}^{m} = \sum\nolimits_{w \in W} {\sum\nolimits_{r \in R} {\sum\nolimits_{m \in M} {\delta_{a}^{r,w} f_{r,w}^{m} } } } } & {\quad \forall a \in A,m \in M,r \in R} \\ \end{array}$$

where ${f}_{r,w }^{m}$ denotes the route $r$ traffic flow of travel model $m$ for OD $w$. Besides, the OD travel demand is expressed as:

5 $$ \sum\nolimits_{{r \in R_{w}^{m} }} {f_{r,w}^{m} = q_{w}^{m} } { }\quad \forall w \in W,m \in M$$

where ${q}_{w}^{m}$ denotes the travel demand of the travel model $m$ for OD $w$.

### Mathematical model

#### Optimal objective

To investigate the interdependent relationship between travel efficiency, emissions, and construction budget in the context of mixed traffic flow, we consider the system travel cost, CO_2_ emissions, and lane construction cost as the optimal objectives.

1. System travel cost
  There are two types traffic flow in the network. Therefore, the system travel cost ${O}_{T}$ equals the sum of the costs of the two types of traffic flows.
  6 $${O}_{T}={O}_{H}+{O}_{F}$$
  Subject to:
  7 $$ O_{H}  = \mathop \sum \limits_{{a \in A}} c_{a}^{H} \left( {v_{a}^{H} } \right)\quad \forall a \in A  $$
  8 $$ O_{F}  = \sum\nolimits_{{a \in A}} {c_{a}^{F} \left( {v_{a}^{F} } \right)} \quad \forall a \in A $$
  9 $$  c_{a}^{H}  = \kappa ^{H} t_{a} \left( {v_{a} } \right) + \Lambda ^{H} l_{a} ~\quad \forall a \in A $$
  10 $$  c_{a}^{F}  = \kappa ^{F} t_{a} \left( {v_{a} } \right) + \Lambda ^{F} l_{a} ~\quad \forall a \in A $$
  where ${O}_{HV}$ and ${O}_{FV}$ represent the system travel time cost of HFCVs and FVs, respectively; ${c}_{a}^{H}$ and ${c}_{a}^{F}$ denote the travel time cost of link $a$ for HFCVs and FVs; ${\kappa }^{H}$ and ${\kappa }^{F}$ denote the value of time (VOT) for HFCVs and FVs. Given HFCV users have different perceptions of environmental costs as regards integrating emissions-related HFCV user costs into generalized link costs. Equation (9) is applied to address the travel cost of HFCV users^36^. The ${\Lambda }^{m}$ is recommended to capture the environmental awareness of HFCV users, where the different ${\Lambda }^{m}$ values indicate different representations of environmental cost.
2. CO_2_ Emission
  The CO_2_ emissions of hydrogen fuel cell vehicles (HFCVs) is expressed as Eq. (11)^11^. Notably, the driving process of HFCVs does not produce CO_2_ emissions. Instead, CO_2_ emissions stem mainly from hydrogen gas production, which we compute using Eq. (12) for fossil fuel vehicles (FVs)^37^. Consequently, we formulate the total CO_2_ emission (g) using Eq. (13).
  11 $$ e_{{a,{\text{~}}H}} \left( {v_{a} } \right) = l_{a} \tau \quad \forall a \in A $$
  12 $$ e_{{a,~F}} \left( {\nu _{a} } \right) = l_{a} 3158\nu ^{{ - 0.56}} \quad \forall a \in A   $$
  13 $$    O_{e}  = \sum\nolimits_{{a \in A}} {(e_{{a,~H}} \left( {t_{a} } \right)}  + e_{{a,~F}} \left( {\nu _{a} } \right)\quad \forall a \in A  $$
  where ${l}_{a}$ denote the length of link $a$; $\tau$ is the CO_2_ emissions per unit distance of HFCV, $\tau =178$ g; ${\upnu }_{a}$ is the average speed on the link $a$.
3. Construction cost
  Balancing construction cost and road network efficiency is crucial for sustainable network design when budget is constrained. The system construction cost is equivalent to the sum of all lane costs in the network.
  14 $$ O_{B}  = \sum\nolimits_{{a \in A}} {\sum\nolimits_{{i \in I_{a} }} {u_{i} d_{i} {\text{~}}y_{i} } } \quad \forall i \in I_{a} ,{\text{~}}a \in A $$
  15 $$ {{\text{Subject to}}:}  $$
  16 $$ y_{i}  \in \left\{ {0,1} \right\}\quad \forall i \in I_{a}  $$
  17 $$ \sum\nolimits_{{a \in A}} {\sum\nolimits_{{i \in I_{a} }} {y_{i} I_{a}  \le B} } {\text{~}}\quad \forall {\text{~}}i \in I_{a} ,a \in A   $$
  where ${u}_{i}$ is the construction unit cost of lane $i$; ${d}_{i}$ is the length of lane $i$; ${y}_{i}$ is the binary variable, if ${y}_{i}$=1, the lane $i$ is selected to deploy, otherwise ${y}_{i}$=0; ${I}_{a}$ is the candidate lane set; $B$ is the total budget for lane construction.

#### Multiclass network equilibrium

This paper adopts the Wardrop user equilibrium (UE) principle to model user travel choice^38^. Consequently, the mixed traffic flow network equilibrium is represented as non-linear complementarity constraints. That is,

18 $$ f_{{r,w}}^{m} \left( {c_{{r,w}}^{m}  - {\mathcal{C}}_{w}^{m} } \right) = 0~\quad \forall m \in M,~r \in R,w \in W     $$

19 $$ c_{{r,w}}^{m}  - {\mathcal{C}}_{w}^{m}  \ge 0\quad \forall m \in M,{\text{~}}r \in R,w \in W $$

20 $$ \sum\nolimits_{{r \in R_{w}^{m} }} {f_{{r,w}}^{m}  = q_{w}^{m} } \quad \forall m \in M,{\text{~}}r \in R_{w}^{m} ,w \in W $$

21 $$ f_{{r,w}}^{m}  \ge 0{\text{~}}\quad \forall m \in M,{\text{~}}r \in R,w \in W  $$

where ${c}_{r,w}^{m}$ denotes the travel cost of route $r$ for the OD pair $w$; ${\mathcal{C}}_{w}^{m}$ is the minimum travel time of travel model $m$ for OD pair w;${R}_{w}^{m}$ denotes the travel route for OD pair $w\in W$ of vehicle type $m$.

#### Optimization model

Based on the analyses presented in sections “The proposed multi-objective PSO algorithm” and “The diagonalized Frank-Wolfe algorithm”, we propose a multi-objectives optimization model that can be formulated as follows:

22 $$ \mathop {\min }\limits_{y} \left[ {O_{T} \left( {t_{a} \left( {v_{a} } \right),y} \right),O_{e} \left( {t_{a} \left( {v_{a} } \right),y} \right),O_{B} \left( y \right)} \right] $$

Subject to:

23 $$ y_{i}  \in \left\{ {0,1} \right\}\quad \forall i \in I_{a}  $$

24 $$ \sum\nolimits_{{a \in A}} {\sum\nolimits_{{i \in I_{a} }} {y_{i} I_{a}  \le B} } \quad \forall {\text{~}}i \in I_{a} ,a \in A   $$

25 $$ C_{a}  = \mathbb{C}_{a}  + \sum\limits_{{i \in I_{a} }} {y_{i} } \mathbb{C}_{a} \quad \forall ~a \in A $$

26 $$ f_{{r,w}}^{m} \left( {c_{{r,w}}^{m}  - {\mathcal{C}}_{w}^{m} } \right) = 0{\text{~}}\quad \forall m \in M,{\text{~}}r \in R,w \in W  $$

27 $$ c_{{r,w}}^{m}  - {\mathcal{C}}_{w}^{m}  \ge 0{\text{~~}}\quad \forall m \in M,{\text{~}}r \in R,w   $$

28 $$  \sum\nolimits_{{r \in R}} {f_{{r,w}}^{m}  = q_{w}^{m} {\text{~}}} {\text{~}}\quad \forall m \in M,{\text{~}}r \in R,w \in W $$

29 $$ f_{{r,w}}^{m}  \ge \quad \forall m \in M,{\text{~}}r \in R,w \in W $$

where the objective function of the proposed model aims to identify the multi-objective optimal solution. The 0–1 variable constraint is represented by Constraint (23), which serves as the decision variable. Constraint (24) is the budget constraint. Constraint (25) reflects the capacity under different network design schemes, while Constraints (26)–(29) represent the multi-class user equilibrium conditions.

## Solution algorithm

The proposed multi-objective bi-level programming model with equilibrium constraints belongs to the scope of the NP-hard (Non-deterministic Polynomial hard) problem. The upper-level aim is to find the optimal network design scheme. The lower-level problem describes travel route choice, i.e., the multi-class traffic assignment, which is describe as nonlinear complementary constraints. A comprehensive solution framework that combines the PSO algorithm and the Frank-Wolfe algorithm is developed to solve the established mathematical model.

### The proposed multi-objective PSO algorithm

Particle Swarm Optimization (PSO) is a type of Swarm Intelligence (SI) algorithm that utilizes group cooperation and simulates the foraging behavior of birds to perform random searches, which was originally proposed by Eberhart and Kennedy^39^. PSO can effectively utilize the optimal individuals in the population and their information, guiding the evolution process and considering individual personality. It has become a popular research topic in the field of intelligent optimization algorithms, which also has been widely applied in the transportation field^40,41^. However, the basic PSO algorithm fail to handle discrete variable and multi-objective optimization problems. Hence, we developed a multi-objective PSO algorithm (MPSO), which incorporates a multi-objective non-dominated solution ranking and a local search function with variable neighborhood structure. Fast non-dominated solution ranking method first developed in the NSGA-II is efficiency algorithm to find the Pareto solution set. Three strategies are designed to improve the ranking efficiency: (1) Fast non-dominated sorting operator, which is used to stratify the Pareto solution set; (2) The crowding distance is proposed, which is used to rank the solution of the same class; (3) Selection operator with elite strategy, which is used to find the optimal solutions by iteration. The specific explanation for the above strategies can ref^42^. Here, the fast non-dominated solution ranking algorithm is adopted to MPSO.

**Figure 2:**
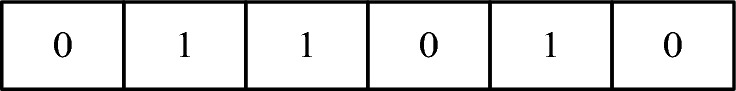
A design scheme of the link.

**Figure 3:**
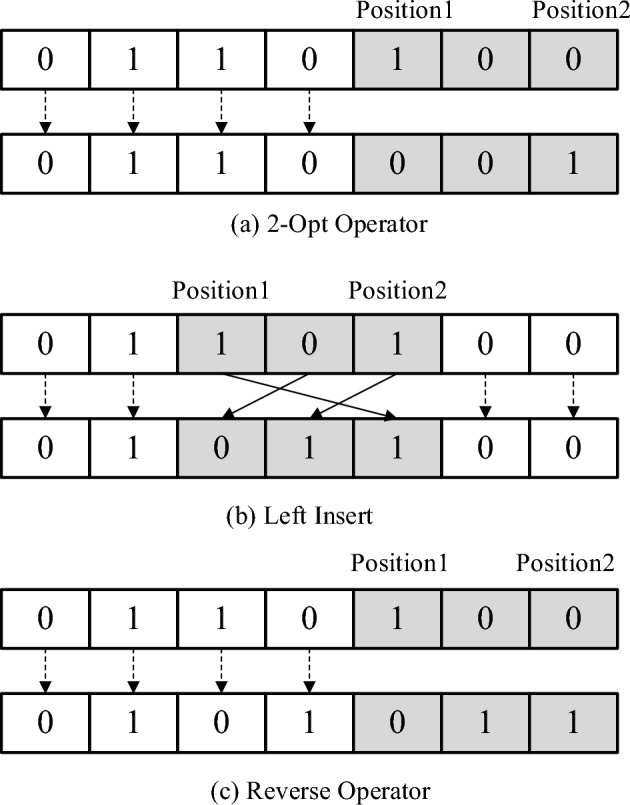
Neighborhood structure.

In this paper, we adopted 0–1 encoding method to represent the candidate road link. In detailed, the design scheme of the road network links is represented as a discrete row vector with elements of 0–1 variables, as shown in Fig. 2.

where the column indicates the candidate set size, and Fig. 2 indicates the $\widehat{A}$ includes six candidate links. The elements are that the link is selected for modification is denoted as 1 variable, 0 otherwise. Further, we propose to replace the particle update process in the base PSO algorithm with a variable neighborhood search (VNS) operator. VNS is an improved local search algorithm. It uses the neighborhood structure composed of different actions to perform alternating searches. There are three neighborhood structure to conduct a local search, as shown in Fig. 3.

1. 2-opt operator: Selects two random links (Positions 1 and 2) in the scheme and rotation the scheme between the selected links (Fig. 3a).
2. Left Insert operator: Randomly selects a link (Position 1) in the scheme, and inserts link 1 to a selected position (Position 2) on the left of Position 2 (Fig. 3b).
3. Reverse operator: Selects two random links (Positions 1 and 2) in the scheme and perform the 0–1 value transformation between the selected links (Fig. 3c).

In basic POS algorithm, the position and velocity equations are used to update the particles in the form of continuous variables. In this work, we employ the neighborhood structure to perform the update process. The detailed steps of MPSO algorithm are in Table 1.

**Table 1:** The detailed steps of MPSO algorithm.

MPSO algorithm	
Input	Set $N$ ; The basic MPSO Algorithm parameters:${G}_{max}$
Step 0	*Initialization*. Randomly generate an initial solution set of size $N$
Step 1	*Fitness calculation*. Calculating the objectives value of the initial solutions set
Step 2	*Finding non-inferior solution set*. Applying non-dominated solution sorting algorithm to find non-inferior solution set
Step 3	*Updating.* Performing VNS algorithm to update the solution set based on the neighborhood structure and conduct fitness calculation for the solution set
Step 4	*Update the non-inferior solution set*. Updating the non-inferior solution set based on the new solutions set
Step 5	*Convergence test.* If the maximum generation (${G}_{max}$) is achieved, go to Step 6; otherwise, go to Step 1
Step 6	*Output.* Obtaining Pareto Frontier

### The diagonalized Frank-Wolfe algorithm

The mixed traffic flow consisting of HVs and CAVs is hard to solve due to the asymmetric influence of the two types of traffic flows. In this paper, we employ a diagonalized Frank-Wolfe algorithm to address the multiclass equilibrium problems. The algorithm has adopted to handle mixed traffic flow by many studies^43^. The diagonalization algorithm divides the multiuser assignment problem into a number of sub-problems. The detailed steps of the diagonalization algorithm are listed in Table 2^44^.

**Table 2:** The detailed steps of the diagonalization algorithm.

Diagonalization algorithm	
Step 0	*Initialization*. Find a feasible link-flow pattern vector, ${{\varvec{x}}}_{n}$. Set *n*: = 0
Step 1	*Diagonalization*. Perform one iteration of the convex combinations algorithm on the diagonalized subproblem (e.g., Frank-Wolfe Algorithm), using ${{\varvec{x}}}_{n}$ as the initial solutions. This yields a link-flow pattern ${{\varvec{x}}}_{n+1}$
Step 2	*Convergence test*. If ${{\varvec{x}}}_{n+1}\cong {{\varvec{x}}}_{n}$ , stop. If not *n*: = *n* + 1, go to Step 1

In the Table 2, The subproblems is defined as a single user equilibrium, which is solved by Frank-Wolfe algorithm. The detailed steps of the Frank-Wolfe algorithm are shown in Table 3 as follows:

**Table 3:** The detailed steps of the Frank-Wolfe Algorithm.

Frank-Wolfe algorithm	
Input	Network $G \left(N, A\right)$, the network design scheme, $\varepsilon$
Step 0	*Initialization*. Based on the network design scheme, conducting all-or-nothing assignments for HFCV or FV in the network
Step 1	*Update link travel time*. That is, updating the ${t}_{a}\left({v}_{a}\right)$ according to the all-or-nothing assignment
Step 2	*Determining the next search direction*. Conducting all-or-nothing assignments again depending on the updated the ${t}_{a}\left({v}_{a}\right)$ and obtaining the auxiliary flow ${y}_{a}^{n}$
Step 3	*Calculating the iteration step size*. Calculating the $\lambda$ in the following equation using the dichotomous method $\sum_{a}({y}_{a}^{n}-{x}_{a}^{n}){t}_{a}\left[{x}_{a}^{n}+\lambda ({y}_{a}^{n}-{x}_{a}^{n})\right]=0$
Step 4	*Determining new search direction by the following equation:* ${{x}_{a}^{n+1}=x}_{a}^{n}+\lambda ({y}_{a}^{n}-{x}_{a}^{n})$
Step 5	*Convergence test*. If $\frac{\sqrt{\sum_{a}{({x}_{a}^{n+1}-{x}_{a}^{n})}^{2}}}{\sum_{a}{x}_{a}^{n}}\le \varepsilon$ , stop. $\varepsilon$ is the convergence accuracy. If not *n*: = *n* + 1, go to Step 1

## Numerical experiments

### Nguyen-Dupius network

**Figure 4:**
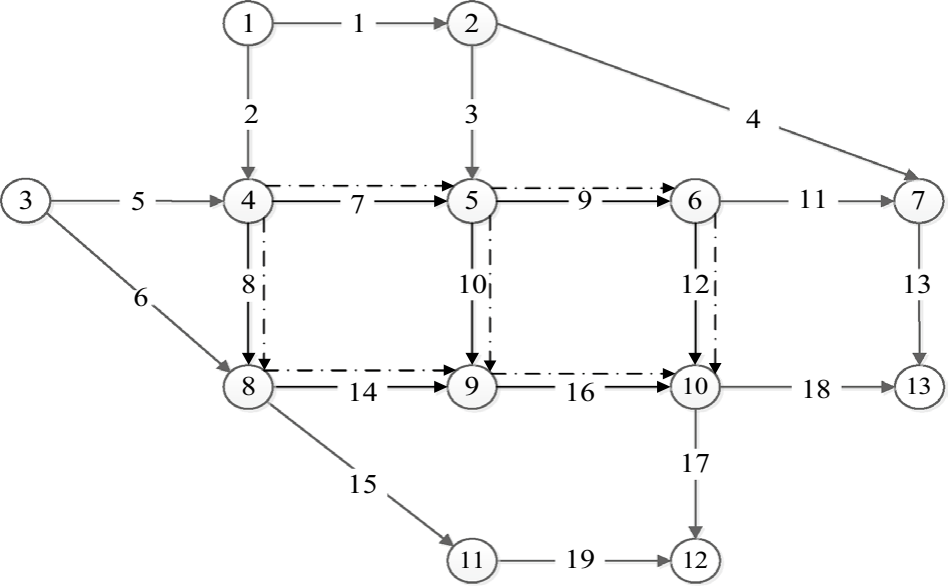
Nguyen-Dupuis networks.

1. Base scenario
  In this section, we demonstrate the effectiveness of the proposed model and algorithm by applying to the Nguyen-Dupius network (Fig. 4). The network comprises 13 nodes, 19 links, and 4 OD (origin–destination) pairs. The attributes of the links are listed in detail in Table 4^6^. The seven dashed lines indicate the candidate links. In base scenario, we assume that the penetration rate of HFCVs is assumed to be 50%. The other parameters are set as: $\tau =178$ g;${u}_{i}=\mathrm{10,0000} \$$; $\alpha =0.15$; $\beta =4$; ${\kappa }^{H}={\kappa }^{H}=0.5 \$/{\text{min}}$;${\Lambda }^{H}=0.5\$$; ${\Lambda }^{F}=1.5\mathrm{\$}$. Table 5 show the detailed comparison results between MPSO and NSGA-II. Compared with NSGA-II, MPSO has an advantage over genetic algorithms in terms of computational efficiency. However, in Scenario 1, MPSO obtains fewer Pareto solutions compared to genetic algorithms. Based on the comparison results in Table 5, we can conclude that the MPSO is effective in solving the our problem.

**Table 4:** Link attributes of modified Nguyen-Dupius network.

Link	${t}_{a}^{0}$	#Lanes	Capacity	Length	Link	${t}_{a}^{0}$	#Lanes	Capacity	Length
1	9	3	3000	5.4	11	2	3	3000	3
2	7	4	4000	4.2	12	9	4	4000	5.4
3	7	3	3000	4.2	13	9	3	3000	5.4
4	14	2	2000	8.4	14	10	4	4000	6
5	9	3	3000	5.4	15	9	2	2000	5.4
6	12	3	3000	7.2	16	6	4	4000	3.6
7	3	4	4000	1.8	17	5	3	3000	3
8	9	3	3000	5.4	18	9	4	4000	5.4
9	5	3	3000	3	19	11	3	3000	6.6
10	13	3	3000	7.8

**Table 5:** Comparison results between MPSO and NSGA-II.

Scenarios	*g*_*max*_	*Popsize*	Method	Running time (s)	Solution no
1	5	10	MPSO	185.4	8
			NSGA-II	187.3	9
2	5	20	MPSO	318.2	15
			NSGA-II	323.8	15
3	10	10	MPSO	320.5	9
			NSGA-II	325.3	9
4	10	20	MPSO	572.8	15
			NSGA-II	580.4	15

In this paper, we propose three scenarios to compare and analyze the impact of road network design considering HFCVs on the transportation system. Scenario 1 (S1) does not consider the introduction of HFCVs and without conduct road network design. Scenario 2 (S2) considers HFCVs and without conduct road network design. Scenario 3 (S3) not only considers the existence of HFCVs but also performs the road network design. Table 6 shows the objectives value of three scenarios. It is worth noting that the objective value in S3 is the mean of multiple Pareto solutions.

**Table 6:** The objective value for the three scenarios.

	S1	S2	S3
Total emission (kg)	297,892	183,297	168,179
Total travel cost ($)	2,131,900	1,705,600	1,397,127

**Figure 5:**
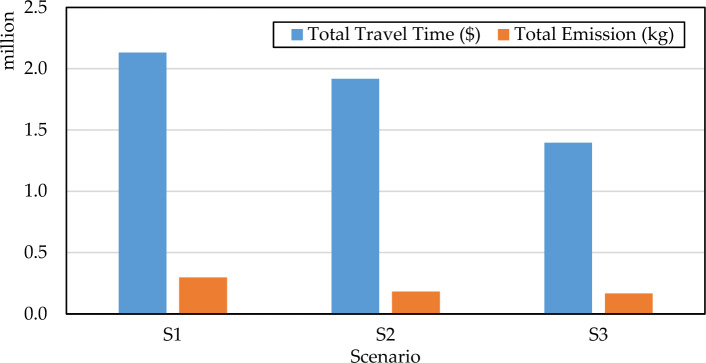
Total travel cost and total CO_2_ emission of three scenarios.

**Figure 6:**
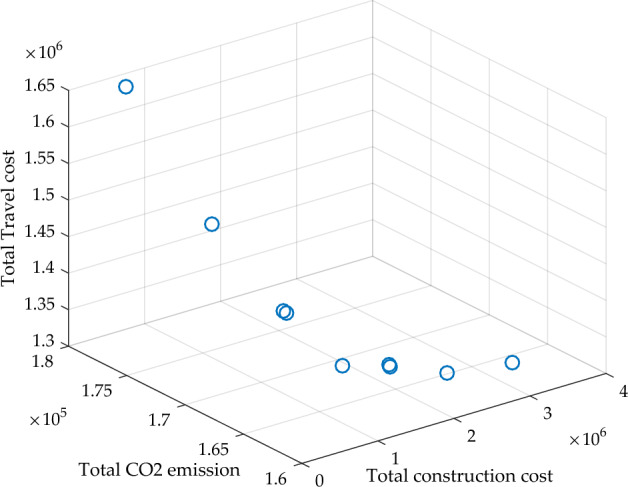
Pareto results of the three-objective value.

Figures 5 and 6 provide a visual representation of the optimization results. Based on Table 6 and Fig. 5, we know that in S2, total CO_2_ emission cost and total travel costs decreased by 62.5% and 20%, respectively, compared with S1. It reveals that HFCVs will dramatically decrease the transportation system emission. More actions should adopt to promote the development of HCFVs. Compared with S1, total CO_2_ emission cost, and total travel costs decreased by 77.1% and 52.6%, respectively under S3. This shows that a better road network design has a good effect on improving the transportation system performance, although it need pay some construction costs. Compared with S2, total CO_2_ emission cost, and total travel costs decreased by 8.2% and 22.1%, respectively under S3. The results show that total travel costs and travel are also reduced. Thus, we can conclude that more HCFVs and reasonable road network design are future benefit to reducing the travel cost and CO_2_ emission. From the perspective of transportation managers, a rational road network planning strategy for the emergence of HFCVs can help reduce system emissions. To better evaluate the system performance, a sensitivity analysis was performed for different parameters, e.g., HFCV penetration, in next section.

**Figure 7:**
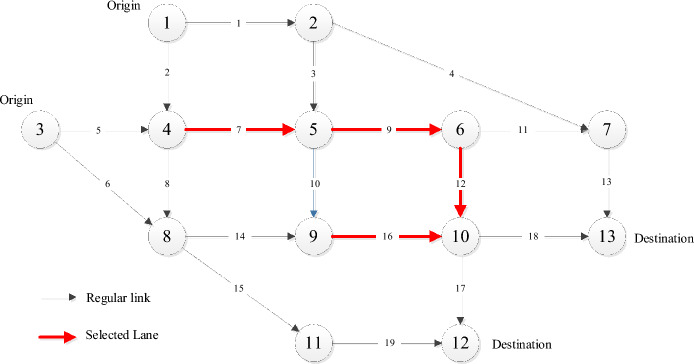
A visual diagram of the optimal scheme (Solution 3 from Table 7).

Table 7 and Fig. 7 show the detailed schemes. Table 7 lists the detailed locations of lanes need to redesigned for all the Pareto solutions. To have a visual presentation, we draw Fig. 7 to show the specific lanes locations that takes the solution 3 as example (Table 7).

**Table 7:** Pareto fronts solution of the Nguyen-Dupius network.

Solution number	Lanes location						
1	4–5	4–8	5–6	5–9	6–10	8–9	9–10
2	4–8	5–6					
3	4–5		5–6		6–10		9–10
4	4–8	5–6				9–10	
5	4–5		5–6		6–10		
6	4–5	4–8	5–6		6–10	8–9	9–10
7	4–5	4–8	5–6			8–9	9–10
8	4–5	4–8	5–6		6–10		9–10
9	4–5				6–10

**Figure 8:**
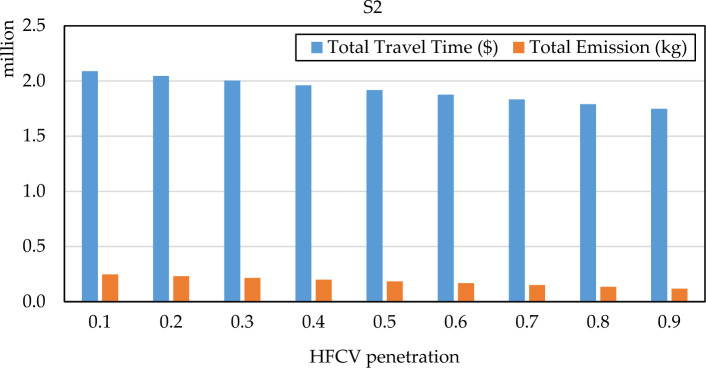
The system performance of S2 under different HFCV penetration rate.

**Figure 9:**
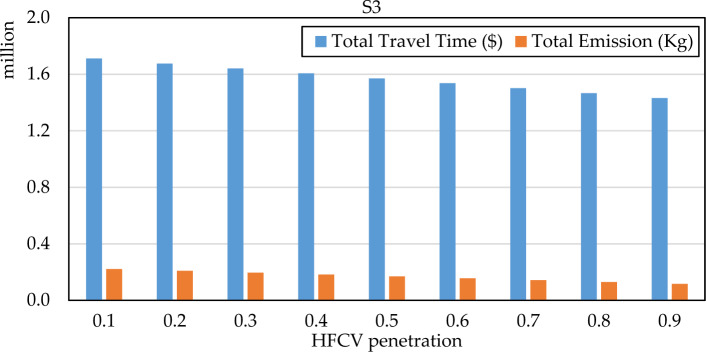
The system performance of S3 under different HFCV penetration rate.
  2. Vehicle speed
    As an important parameter, the driving speed of vehicles will greatly affect the transportation network performance. Thus, we investigate total travel cost and total CO_2_ emission at different vehicle speeds under scenario 3, which are shown in Fig. 10. Based on Fig. 10, we know that vehicles traveling at higher speeds are benefit for the environment, in other words, higher vehicle speed will produce less CO_2_ emissions. Another notable finding is that as the vehicle speed increases, the total travel cost of the system will also increase. The reason is that the total travel cost depends on the BPR function. A higher travel speed will decrease the travel time. The results remind us that the vehicle speed of the vehicle will affect the total emissions and total travel costs. Therefore, it is also a feasible way to reduce system emissions by improving driving speeds. Figure 11 further presents the pareto frontiers under different vehicle speeds, which also proves that high speed driving can reduce vehicle emissions and travel expenses.

(2) Sensitivity analysis
  1. HFCV penetration
    The HFCV penetration rate reflects the market share of HFCV vehicles and illustrates the extent to which HFCV technology has evolved. Figures 8 and 9 show the total CO_2_ emission and total travel cost considering different HFCV penetration rates. We can find that the total travel cost decrease, and the total CO_2_ emission also decrease as the penetration rate increases. The results indicate that more HFCVs will benefit the system emission, which helps decision-makers to formulate the relevant policy to promote HFCVs adoption. The total travel cost also decreases because of the different travel cost values and environmental cost between HFCV and FV. In other word, HFCV have lower emission travel cost.

**Figure 10:**
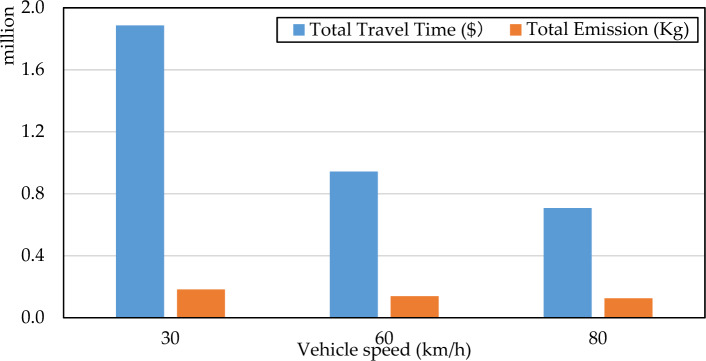
The system performance under different vehicle speed.

**Figure 11:**
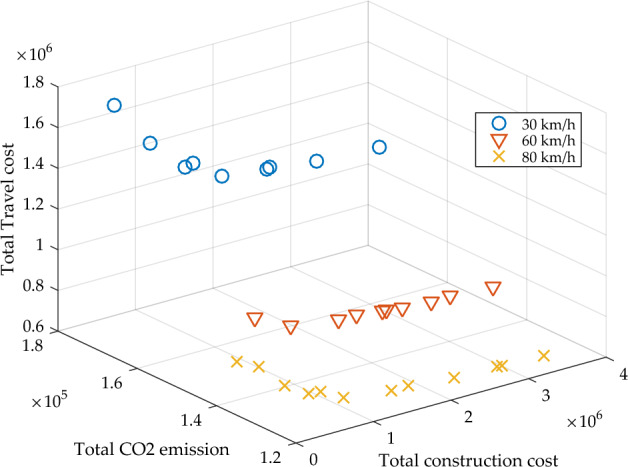
Pareto frontiers under different vehicle speed.
  3. Travel demand
    Travel demand can greatly influence the performance of the transportation network. Analyzing the performance of the road network under different travel demands facilitates the development of rational policies. Hence, we present the total travel cost and total CO_2_ emission of scenario 3 under three demand level: base demand, 2 × demand, and 3 × demand. Figure 12 shows the detailed results of three demand level. Base on Fig. 12, compared to base demand, the travel CO_2_ emission of 2 × demand increased almost 4 times and increased 20 times of 4 × demand. The same disproportionate characteristic is seen in total travel time. This reveals that total travel time will increase rapidly with travel demand because of the nonlinearity of BPR. In other words, we cannot intuitively assume that there is a proportional relationship between demand and road network performance. Figure 13 presents the detailed frontier of S3 under different demand.

**Figure 12:**
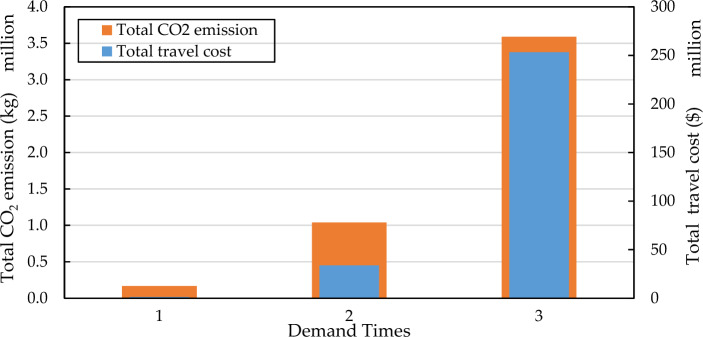
System performance under different travel demand level.

**Figure 13:**
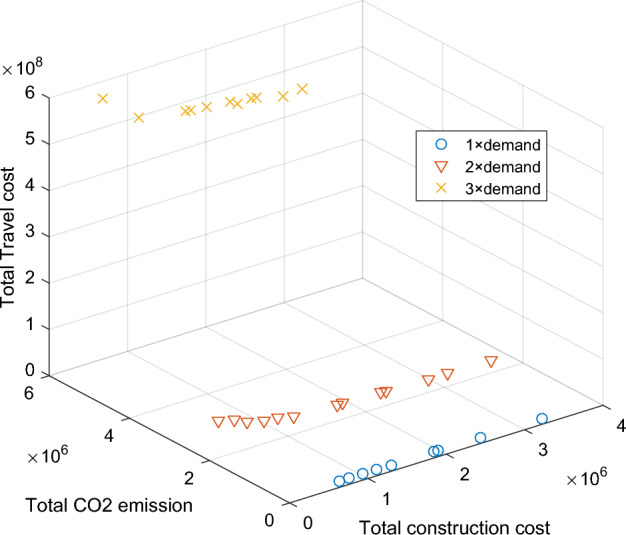
Pareto frontiers under different travel demand.

### The Sioux Falls network

To further investigate the system performance with HFCVs, we applied the model to the Sioux Falls network. The Sioux Falls network (Fig. A1), consisting of 24 nodes, 76 regular links Tables B1 and B2 shows the OD demand^6^. The rest of the parameters are consistent with the Nguyen-Dupius network.

Based on Table 8, we know that in S2, total CO_2_ emission cost and total travel costs decreased by 12.3% and 11.5%, respectively, compared with S1. It also further demonstrates that HFCVs can decrease the transportation system emission. Compared with S1, total CO_2_ emission cost, and total travel costs decreased by 13.5% and 14.9%, respectively under S3. Similarly, compared with S2, total CO_2_ emission cost, and total travel costs decreased by 1.3% and 3.8%, respectively under S3. The results also show that total travel costs and travel emission are also reduced. Therefore, we are further convinced that the emergence of HFCV and road network design can largely contribute to the development of sustainable transportation. Comparing the two networks, we also find that the network structure has a significant impact on the target results, as further demonstrated by the results above. The different results resulting from the variability of network topologies also remind us to examine the structure of the road network when formulating different planning policies.

**Table 8:** The objective value for the three scenarios.

	S1	S2	S3
Total emission (kg)	392,003	343,628	339,123.1
Total travel cost ($)	1,934,239.6	1,712,272.4	1,646,517.9

## Conclusions

In this study, we propose a bi-level multi-objective programming model that considers economic and environmental sustainability to address sustainable network design problems with HFCVs. The upper level aims to identify an optimal network design scheme, while the lower level addresses the multiclass traffic flow assignment consisting of HFCVs and FVs. To achieve multi-objective sustainable network design, we develop an integrated solution framework that combines the MPSO algorithm and the diagonalized Frank Wolfe algorithm. We demonstrate the effectiveness and efficiency of our model and algorithm by applying them to the Nguyen-Dupius network and Sioux Falls network. The results indicate that total CO_2_ emission costs and total travel costs will be decreased based on network design and the emergency of HFCV. The sensitivity analysis shows that as the HFCV penetration rate increases, total travel costs and CO_2_ emissions decrease. Additionally, higher vehicle speed and travel demand also decrease emissions and total travel costs. In conclusion, the development of HFCVs and reasonable road network design can contribute to the sustainable transportation development.

Several issues merit further study in the future. Firstly, the growth in demand for HFCVs remains uncertain, and there is currently a lack of applicable demand forecasting models. Secondly, the impact of autonomous driving technology on HFCV emissions should be further investigated, as it is developing rapidly. Finally, a more precise algorithm for solving the bi-level model is worth exploring, such as decomposition algorithms or a linearization-based solution approach.

### Supplementary Information

Supplementary Information.

## Data Availability

All data generated or analyzed during this study are included in this published article. The codes during the current study are not publicly available but are available from the corresponding author on reasonable request.

## Abbreviations

HFCV: Hydrogen fuel cell vehicle
FV: Fuel vehicle
$N$: Set of nodes
$A$: Set of links
$M$: Type of vehicles ($m\in M$); $M=\left\{{\text{HFCV}},\mathrm{ FV}\right\}$
$R$: Set of paths
${C}_{a}$: Capacity of link $a$
$${\mathbb{C}}_{a}$$: Lane capacity
$${y}_{a}$$: Binary variable
${D}^{w}$: Travel demand of OD $w$
${l}_{a}$: Length of link $a$
${f}_{w,m}^{r}$: Traffic flow on path $r$
${t}_{a}$: Travel time on link $a$
$${c}_{w,m}^{r}$$: Travel cost
${\mathcal{C}}_{w}^{m}$: The Minimum travel cost
${v}_{a}$: Traffic flow on link $a$
${e}_{a, H}$: The CO_2_ emission of HFCV
${e}_{a, F}$: The CO_2_ emission of FV
${\upnu }_{a}$: The average speed on the link $a$
${u}_{i}$: The construction unit cost of lane $i$
$\tau$: The unit emission

## References

[1] Alves J, Baptista PC, Gonçalves GA, Duarte GO (2016). Indirect methodologies to estimate energy use in vehicles: Application to battery electric vehicles. Energy Convers. Manag..

[2] Wang F, Yan J (2022). CO_2_ storage and geothermal extraction technology for deep coal mine. Sustainability.

[3] Su C, Urban F (2021). Carbon neutral China by 2060: The role of clean heating systems. Energies.

[4] Peyravi B, Peleckienė V, Vaičiūtė K (2022). Research on the impact of motorization rate and technological development on climate change in lithuania in the context of the European green deal. Sustainability.

[5] Manoharan Y (2019). Hydrogen fuel cell vehicles; Current status and future prospect. Appl. Sci..

[6] Lin Y (2021). Multiobjective environmentally sustainable optimal design of dedicated connected autonomous vehicle lanes. Sustainability.

[7] Zhang Q, Chen W, Ling W (2022). Policy optimization of hydrogen energy industry considering government policy preference in China. Sustain. Production Consum..

[8] Wang T (2022). Prediction of the impact of meteorological conditions on air quality during the 2022 Beijing winter olympics. Sustainability.

[9] Ajanovic A, Haas R (2021). Prospects and impediments for hydrogen and fuel cell vehicles in the transport sector. Int. J. Hydrog. Energy.

[10] Zhang X, Qiu G, Wang S, Wu J, Peng Y (2022). Hydrogen leakage simulation and risk analysis of hydrogen fueling station in China. Sustainability.

[11] Gallo M, Marinelli M (2022). The impact of fuel cell electric freight vehicles on fuel consumption and CO_2_ emissions: The case of Italy. Sustainability.

[12] Yang H (2022). Exploring future promising technologies in hydrogen fuel cell transportation. Sustainability.

[13] Jia H, Lin Y, Luo Q, Li Y, Miao H (2019). Multi-objective optimization of urban road intersection signal timing based on particle swarm optimization algorithm. Adv. Mech. Eng..

[14] Frank M, Wolfe P (1956). An algorithm for quadratic programming. Naval Res. Logist. Q..

[15] Evangelisti S, Tagliaferri C, Brett DJL, Lettieri P (2017). Life cycle assessment of a polymer electrolyte membrane fuel cell system for passenger vehicles. J. Cleaner Production.

[16] Bethoux O (2020). Hydrogen fuel cell road vehicles and their infrastructure: An option towards an environmentally friendly energy transition. Energies.

[17] Offer GJ, Howey D, Contestabile M, Clague R, Brandon NP (2010). Comparative analysis of battery electric, hydrogen fuel cell and hybrid vehicles in a future sustainable road transport system. Energy Policy.

[18] Zhao F (2020). Hydrogen fuel cell vehicle development in China: An industry chain perspective. Energy Tech..

[19] Tao Y, Qiu J, Lai S, Zhang X, Wang G (2020). Collaborative planning for electricity distribution network and transportation system considering hydrogen fuel cell vehicles. IEEE Trans. Transp. Electrific..

[20] Ahmadi P, Kjeang E (2015). Comparative life cycle assessment of hydrogen fuel cell passenger vehicles in different Canadian provinces. Int. J. Hydrog. Energy.

[21] Cai Z, Ou X, Zhang Q, Zhang X (2012). Full lifetime cost analysis of battery, plug-in hybrid and FCEVs in China in the near future. Front. Energy.

[22] Li Y, Chen DW, Liu M, Wang RZ (2017). Life cycle cost and sensitivity analysis of a hydrogen system using low-price electricity in China. Int. J. Hydrog.en Energy.

[23] Sun H (2017). Hydrogen station siting optimization based on multi-source hydrogen supply and life cycle cost. Int. J. Hydrog. Energy.

[24] Ogden, J. M., Williams, R. H. & Larson, E. D. Societal lifecycle costs of cars with alternative fuels/engines. *Energy Policy* **22** (2004).

[25] Xu X, Chen A, Yang C (2016). A review of sustainable network design for road networks. KSCE J. Civ. Eng..

[26] Wang Y, Szeto WY (2017). Excessive noise paradoxes in urban transportation networks. Transp. A Transport Sci..

[27] Sharma S, Mathew TV (2011). Multiobjective network design for emission and travel-time trade-off for a sustainable large urban transportation network. Environ. Plan. B Plan. Des..

[28] Ma R, Ban X, Szeto WY (2015). Emission modeling and pricing in dynamic traffic networks. Transp. Res. Procedia.

[29] Wang H, Lam WHK, Zhang X, Shao H (2015). Sustainable transportation network design with stochastic demands and chance constraints. Int. J. Sustain. Transp..

[30] Banister D (2007). Sustainable transport: Challenges and opportunities. Transportmetrica.

[31] Szeto WY, Jaber X, Wong SC (2012). Road network equilibrium approaches to environmental sustainability. Transport Rev..

[32] Acar C, Dincer I (2020). The potential role of hydrogen as a sustainable transportation fuel to combat global warming. Int. J. Hydrog. Energy.

[33] Van Mierlo J, Maggetto G, Lataire Ph (2006). Which energy source for road transport in the future? A comparison of battery, hybrid and fuel cell vehicles. Energy Convers. Manag..

[34] Halder P (2023). Advancements in hydrogen production, storage, distribution and refuelling for a sustainable transport sector: Hydrogen fuel cell vehicles. Int. J. Hydrog. Energy.

[35] Roads, U. S. B. of P. *Traffic Assignment Manual for Application with a Large, High Speed Computer*. (U.S. Department of Commerce, Bureau of Public Roads, Office of Planning, Urban Planning Division, 1964).

[36] Ma J, Cheng L, Li D, Tu Q (2018). Stochastic electric vehicle network considering environmental costs. Sustainability.

[37] Gardner LM, Duell M, Waller ST (2013). A framework for evaluating the role of electric vehicles in transportation network infrastructure under travel demand variability. Transp. Res. Part A Policy Pract..

[38] Wardrop JG, Whitehead JI (1952). Correspondence. Some theoretical aspects of road traffic research. Proc. Inst. Civ. Eng..

[39] Kennedy, J. & Eberhart, R. Particle swarm optimization, in *Proceedings of ICNN’95 - International Conference on Neural Networks*, vol. 4, 1942–1948 (1995).

[40] Wu J, Guo X, Sun H, Wang B (2014). Topological effects and performance optimization in transportation continuous network design. Math. Probl. Eng..

[41] Farahani RZ, Miandoabchi E, Szeto WY, Rashidi H (2013). A review of urban transportation network design problems. Eur. J. Oper. Res..

[42] Deb K, Pratap A, Agarwal S, Meyarivan T (2002). A fast and elitist multiobjective genetic algorithm: NSGA-II. IEEE Trans. Evol. Comput..

[43] Wu Y, Yang H, Zhao S, Shang P (2021). Mitigating unfairness in urban rail transit operation: A mixed-integer linear programming approach. Transp. Res. Part B Methodol..

[44] Daganzo CF, Sheffi Y (1977). On Stochastic models of traffic assignment. Transp. Sci..

