# Supplementary material for: Sustainable road network design considering hydrogen fuel cell vehicles
Source: Sci Rep. 2023 Dec 11;13:21947. doi: 10.1038/s41598-023-49264-1 (PMC10713546; doi:10.1038/s41598-023-49264-1)
Supplement: Supplementary file 1 — Supplementary Information. [file 41598_2023_49264_MOESM1_ESM.docx]

**Appendix A**

**Figure A1.** The Sioux Fall network.

**Appendix B**

**Table B1.** Link characteristics of the Sioux Falls network.

| **Link** | **Time (min)** | **Length** | **Initial Capacity (veh/h)** | **Link** | **Time (min)** | **Length** | **Initial Capacity (veh/h)** |
| --- | --- | --- | --- | --- | --- | --- | --- |
| 1-2 | 6 | 6 | 10,000 | 13-24 | 4 | 4 | 6000 |
| 1-3 | 4 | 4 | 10,000 | 14-11 | 4 | 4 | 4000 |
| 2-1 | 6 | 6 | 10,000 | 14-15 | 3 | 3 | 6000 |
| 2-6 | 8 | 8 | 6000 | 14-23 | 4 | 4 | 4000 |
| 3-1 | 4 | 4 | 10,000 | 15-10 | 6 | 6 | 8000 |
| 3-4 | 4 | 4 | 8000 | 15-14 | 3 | 3 | 6000 |
| 3-12 | 4 | 4 | 10,000 | 15-19 | 3 | 3 | 8000 |
| 4-3 | 4 | 4 | 8000 | 15-22 | 3 | 3 | 8000 |
| 4-5 | 2 | 2 | 8000 | 16-8 | 5 | 5 | 6000 |
| 4-11 | 6 | 6 | 6000 | 16-10 | 4 | 4 | 6000 |
| 5-4 | 2 | 2 | 8000 | 16-17 | 2 | 2 | 6000 |
| 5-6 | 4 | 4 | 4000 | 16-18 | 3 | 3 | 8000 |
| 5-9 | 5 | 5 | 6000 | 17-10 | 8 | 8 | 4000 |
| 6-2 | 8 | 8 | 6000 | 17-16 | 2 | 2 | 6000 |
| 6-5 | 4 | 4 | 4000 | 17-19 | 2 | 2 | 4000 |
| 6-8 | 2 | 2 | 6000 | 18-7 | 2 | 2 | 10,000 |
| 7-8 | 3 | 3 | 6000 | 18-16 | 3 | 3 | 8000 |
| 7-18 | 2 | 2 | 10,000 | 18-20 | 14 | 14 | 10,000 |
| 8-6 | 2 | 2 | 6000 | 19-15 | 3 | 3 | 8000 |
| 8-7 | 3 | 3 | 6000 | 19-17 | 2 | 2 | 4000 |
| 8-9 | 3 | 3 | 4000 | 19-20 | 4 | 4 | 4000 |
| 8-16 | 5 | 5 | 6000 | 20-18 | 14 | 14 | 10,000 |
| 9-5 | 5 | 5 | 6000 | 20-19 | 4 | 4 | 4000 |
| 9-8 | 3 | 3 | 4000 | 20-21 | 6 | 6 | 6000 |
| 9-10 | 3 | 3 | 8000 | 20-22 | 5 | 5 | 6000 |
| 10-9 | 3 | 3 | 8000 | 21-20 | 6 | 6 | 6000 |
| 10-11 | 1 | 1 | 6000 | 21-22 | 2 | 2 | 6000 |
| 10-15 | 6 | 6 | 8000 | 21-24 | 3 | 3 | 4000 |
| 10-16 | 4 | 4 | 6000 | 22-15 | 3 | 3 | 8000 |
| 10-17 | 8 | 8 | 4000 | 22-20 | 5 | 5 | 6000 |
| 11-4 | 6 | 6 | 6000 | 22-21 | 2 | 2 | 6000 |
| 11-10 | 1 | 1 | 6000 | 22-23 | 4 | 4 | 4000 |
| 11-12 | 6 | 6 | 6000 | 23-14 | 4 | 4 | 4000 |
| 11-14 | 4 | 4 | 4000 | 23-22 | 4 | 4 | 4000 |
| 12-3 | 4 | 4 | 10,000 | 23-24 | 2 | 2 | 6000 |
| 12-11 | 6 | 6 | 6000 | 24-13 | 4 | 4 | 6000 |
| 12-13 | 3 | 3 | 10,000 | 24-21 | 3 | 3 | 4000 |
| 13-12 | 3 | 3 | 10,000 | 24-23 | 2 | 2 | 6000 |

**Table B2.** Total origin–destination (OD) demand of the Sioux Falls network (veh/h).

| **O-D** | **Demand** | **O-D** | **Demand** | **O-D** | **Demand** | **O-D** | **Demand** |
| --- | --- | --- | --- | --- | --- | --- | --- |
| 1-6 | 20,000 | 6-15 | 12,000 | 11-1 | 20,000 | 21-7 | 4000 |
| 1-7 | 20,000 | 6-21 | 12,000 | 11-6 | 12,000 | 21-11 | 8000 |
| 1-11 | 20,000 | 7-1 | 20,000 | 11-7 | 20,000 | 21-15 | 12,000 |
| 1-15 | 20,000 | 7-6 | 8000 | 11-21 | 8000 | 15-1 | 20,000 |
| 1-21 | 20,000 | 7-11 | 20,000 | 11-15 | 12,000 | 15-6 | 12,000 |
| 6-1 | 20,000 | 7-21 | 16,000 | 21-1 | 8000 | 15-7 | 12,000 |
| 6-11 | 12,000 | 7-15 | 12,000 | 21-6 | 12000 | 15-11 | 12,000 |
